# Factors deterring dentistry, medical, pharmacy, and social science undergraduates from pursuing nursing as a healthcare career: a cross-sectional study in an Asian university

**DOI:** 10.1186/s12909-018-1118-1

**Published:** 2018-01-26

**Authors:** Ling Ting Wu, Wenru Wang, Eleanor Holroyd, Violeta Lopez, Sok Ying Liaw

**Affiliations:** 1 Alice Lee Centre for Nursing Studies, Yong Loo Lin School of Medicine, National University of Singapore, Level 2, Clinical Research Centre, Block MD11 10 Medical Drive, Singapore, 117597 Singapore; 2 Nursing Research Capacity Building, Aga Khan University, P. O. Box 8842, Kampala, Uganda East Africa

**Keywords:** Career choices, Healthcare, Nursing recruitment, Perceptions of nursing

## Abstract

**Background:**

Globally more registered nurses need to be recruited to meet the needs of aging populations and increased co-morbidity. Nursing recruitment remains challenging when compared to other healthcare programs. Despite healthcare students having similar motivation in joining the healthcare industry, many did not consider nursing as a career choice. This study aims to identify the deterrents to choosing nursing among healthcare undergraduates by examining the differences in the factors influencing healthcare career choices and nursing as a career choice.

**Methods:**

A cross sectional study was conducted using a 35-parallel items instrument known as Healthcare Career Choice and Nursing Career Choice scale. Six hundred and four (*n* = 604) first year medical, pharmacy, dentistry and social science students from a university in Singapore completed the survey.

**Results:**

Nursing as a career was perceived by healthcare students to be more likely influenced by prior healthcare exposure, the nature of the work, job prospects, and social influences. Lack of autonomous decision making, perceived lower ability to make diagnosis, having to attend to patients’ hygiene needs, engendered stigma, and lack of parental support were identified as deterring factors to choosing nursing as a career.

**Conclusion:**

An understanding of the deterrents to choosing nursing as career allows policy makers and educational leaders to focus on recruitment strategies. These include providing more exposure to nurses’ roles in early school years, helping young people to overcome the fear of providing personal hygiene care, promoting nurses’ autonomous nursing practice, addressing gender stigma, and overcoming parental objection.

## Background

Globally, nurses face the greatest workforce demand and yet the most significant challenges in recruiting and retaining nurses [1]. In terms of numbers, nurses formed the largest health care profession. They are also the ones who deliver most direct patient care and the key personnel in managing multiple chronic conditions in all settings [2]. With aging population, nurses’ role has become increasingly crucial and they are now known as the leaders in aging care [3]. Nurses play critical roles in addressing the challenges of the global aging population such as being in the front line of preventive care in the community, acute hospital care, long-term nursing homes as well as assisted living facilities [3]. Owing to nurses’ close proximity to patients and their scientific understanding of care processes across the continuum of care, they are potentially the leaders in improving and redesigning the health care system and its practice environment [4].

However, as healthcare courses such as occupational therapy or physiotherapy continue to increase, nursing was found to be on the losing end in attracting students and was often the last resort career choice [5]. In fact, many countries have reported worsening nursing shortage despite efforts to recruit and retain nurses [1]. In addition, it was found that nursing students are generally found to be older, mainly more than 20 years [6]. This means that the nursing program is facing difficulty in recruiting school leavers such as high school graduates.

A recent systematic review identified the differences in career choice influences among healthcare students. Medical and nursing students were found to have intrinsic factors informing their career decision, whereby a desire to help others was their primary career motivation. On the other hand, dentistry and pharmacy students valued factors such as financial remuneration, job stability, job autonomy, and prestige as important career motivators [7]. Nursing, however, was found to be an unpopular choice among the healthcare courses [8]. Despite school leavers believing that nurses are helpful and caring [9, 10], they expressed no interest in making nursing their career choice. This was found to be highly significant among male students [11]. In general school leavers’ perceptions of nursing were seen to be an “unpleasant job” [12]. Many secondary school students doubted that nursing was a proper degree course due to its low entry requirement when compared to other courses [13]. Moreover, school leavers perceived nursing to have poor social status, involving low skilled ‘dirty’ work such as cleaning others [12], poor financial remuneration [14], low autonomy [10], and lacking in career progression [9].

In Singapore, registered nursing education is offered in two polytechnics and two universities. The polytechnics offer nursing diploma to school leavers who have obtained the General Certificate of Education Ordinary Level (GCE ‘O’ level) while nursing degree offered at universities require school leavers to have obtained the General Certificate of Education Advanced Level (GCE ‘A’ level) or a diploma in related courses. Similar to other countries, nursing courses in Singapore tend to have lower entry requirement to other courses within the same institution in order to meet nursing manpower needs. However, this has led to students believing that nursing is a course for less academic students especially in Singapore where people strongly believe in meritocracy.

A recent qualitative study on healthcare students in Singapore revealed that healthcare students were generally interested in healthcare due to their altruistic personalities or healthcare related experiences. However, students perceived nurses to be less academic, having to perform undesirable task with poor career advancement, and have poor social status [8]. Using these findings, a pair of parallel scales that could measure the differences between influences of healthcare career choices and perceptions of nursing as a career choice was developed and utilized in a previous study. The comparative understanding of career choice influences helped to identify reasons that deter students from choosing nursing as a career [15, 16].

Many studies have explored why students choose nursing and suggest improvements to better attract school leavers [17–19]. These studies mainly address career motivators such as altruism or job stability that attract school leavers to join nursing but rarely considered the barriers to enter a nursing profession. Improvements need to be made to nursing recruitment strategies through understanding what is lacking in a nursing career that attracts school graduates into healthcare careers other than nursing. Hence by identifying the differences in one’s career choice in healthcare as compared to the perception of a nursing career, improvements to nursing recruitment strategies could be made to better attract school leavers.

## Method

### Study aim

This study aims to identify the deterrents to choosing nursing among healthcare undergraduates by examining the differences in factors influencing healthcare career choices and nursing as a career choice.

### Study design

A cross-sectional quantitative study was conducted in National University of Singapore between August to September 2015.

### Participants

This study was conducted via self-administrative questionnaire using an online platform. A population-based sampling method was used to ensure adequate representation of all characteristics of the population [19]. A total of 741 first-year students undertaking medicine (300), pharmacy (175), dentistry (52), and social science (195) courses at one public university in Singapore were recruited.

### Data collection

Following ethical approval by the university’s Institutional Review Board, formal emails were sent to various health care department heads to seek their permissions to recruit their students into this study. The study was conducted at the start of the university’s academic year to ensure that data collection was done when the students were still early in their career decision making process. Students who were interested in participating were asked to log on to the URL link provided in the email to complete the online survey. The participants were informed of their rights to self-determination to ensure that their participation was voluntary. No identifiable information was recorded in completing the questionnaire to ensure the participants’ anonymity.

### Validity and reliability of instrument

A newly developed instrument comprising of two parallel scales, namely the Healthcare Career Choice (HCC) and Nursing as a Career Choice (NCC) scale, was used in this study. This instrument adopted the idea of the parallel scales from an existing Indiana tool with 17 parallel items to determine the differences of career attributes between the nursing career and an ideal career (May et al., 1991). In comparison to the Indiana tool, the HCC_NCC parallel scales examine the differences between factors influencing a healthcare career choice in HCC and perception of the nursing career in NCC. These parallel scales include six subscales such as the ‘personal interest’, ‘prior healthcare exposure’, ‘self-efficacy’, ‘job prospects’, ‘perceived nature of work’ and ‘social influences’, each representing a career attribute that may influence a student’s career decision. The items were developed to be rated on a five-point Likert rating scale (1 = strongly disagree, 2 = disagree, 3 = neutral, 4 = agree, 5 = strongly agree), with higher scores indicating more influential career attribute and lesser scores indicating less influential career attribute.

Psychometric testing of the HCC_NCC parallel scales was established in a previous study [15]. It was validated by 12 content experts, achieving an overall content validation index (CVI) of 0.9. The scale also show great internal consistency reliability with Cronbach’s alpha of more than 0.90 [16]. In this study, both HCC and NCC scales have achieved excellent internal consistency with an overall Cronbach’s alpha of 0.94 and 0.95, respectively.

### Data analysis

Descriptive statistics were used to analyze the demographic data while parametric tests were used to analyze the scales responses. Norman [20] asserts that parametric tests are robust in yielding largely unbiased results when analyzing Likert scales responses. One-way ANOVA was used to examine the differences in career choice influences among the four different healthcare groups. Paired t-test was used to compare parallel items in terms of the differences between the HCC and NCC scale. Significance was set at <0.05.

## Results

### Demographics

A total of 604 out of 741 potential participants responded, which yielded a response rate of 81.5%. The students who participated were a good representation of the number of students in each individual healthcare course. The response rates from each course ranged from 70.9% to 96.4%. Table 1 represents the demographic characteristics of the participants. Most were females (*n* = 380, 62.9%), Singaporeans (*n* = 572, 94.7%), Chinese (*n* = 559, 92.5%), and applied for their university courses after obtaining their General Certificate of Education (GCE) ‘A’ levels (*n* = 512, 84.7%).

**Table 1:** Socio-demographics of participants

Demographics	*N* (%)
Age (years)	
18–21	584 (96.7)
≥ 22	20 (3.3)
Gender	
Male	224 (37.1)
Female	380 (62.9)
Nationality	
(Singaporean)	
Yes	572 (94.7)
No	32 (5.3)
Ethnic group	
Chinese	559 (92.5)
Malay	14 (2.3)
Indian	26 (4.3)
Others	5 (0.9)
Education	
GCE ‘A’ levels	512 (84.7)
Diploma	92 (15.3)
Courses	
Dentistry	47 (7.8)
Medicine	245 (40.6)
Pharmacy	124 (20.5)
Social science	188 (31.1)
Career advice from significant others	
Yes	249 (41.2)
No	355 (58.8)

### Differences between HCC and NCC

**Fig. 1:**
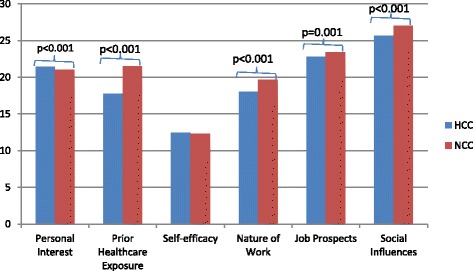
Difference between HCC and NCC in each subscale

Figure 1 illustrates the overall mean scores differences between HCC and NCC in each subscale. Table 2 represents the subscale mean scores differences between HCC and NCC in each healthcare group. Table 3 shows the overall mean score differences between HCC and NCC in each item. The findings were synthesized and elaborated for each subscale.

**Table 2:** Subscales differences between HCC and NCC for each healthcare group

		Medicine			Dentistry			Pharmacy			Social Science		
	Minimum & maximum score	HCC	NCC	t-score	HCC	NCC	t-score	HCC	NCC	t-score	HCC	NCC	t-score
		Mean (±SD)	Mean (±SD)		Mean (±SD)	Mean (±SD)		Mean (±SD)	Mean (±SD)		Mean (±SD)	Mean (±SD)	
Personal interest	(5 items) 5–25	22.70 (±2.63)	21.55 (±3.13)	6.71**	21.60 (±2.93)	20.85 (±3.30)	2.55*	20.62 (±3.25)	20.78 (±2.94)	−053	20.37 (±3.16)	20.50 (±2.75)	−0.576
Prior healthcare exposure	(6 items) 6–30	19.88 (±4.55)	22.24 (±4.17)	−8.53**	19.64 (±4.49)	22.23 (±4.45)	−4.09**	17.50 (±4.50)	21.19 (±3.92)	−9.47**	14.78 (±4.74)	20.48 (±3.44)	−15.90**
Self-efficacy	(4 items) 4–20	12.93 (±3.46)	12.81 (±3.85)	0.46	13.43 (±3.65)	13.14 (±3.50)	0.53	13.08 (±2.55)	12.57 (±3.92)	1.61	11.19 (±3.18)	11.21 (±3.06)	−0.100
Nature of the work	(5 items) 5–25	19.30 (±3.59)	19.51 (±3.23)	−1.01	20.06 (±3.31)	19.78 (±3.11)	0.70	17.90 (±3.35)	19.94 (±2.91)	−6.13**	15.93 (±3.35)	19.57 (±3.31)	−13.03**
Job prospects	(7 items) 7–35	23.58 (±5.86)	23.96 (±5.03)	−1.18	25.38 (±4.50)	24.04 (±4.33)	2.36*	24.16 (±4.95)	23.90 (±4.91)	0.58	20.30 (±5.06)	22.15 (±4.78)	−4.775**
Social influences	(8 items) 8–40	25.99 (±6.37)	27.60 (±5.94)	−4.03**	26.83 (±5.90)	27.66 (±5.24)	−1.13	26.17 (±5.67)	27.08* (±5.15)	−2.02*	24.57 (±5.41)	26.02 (±4.93)	−3.731**

Significant at **p < 0.001, * *p < 0.05*

**Table 3:** Differences between healthcare career choice and nursing as a career choice scores

		HCC		NCC		
Item/Domain		M	SD	M	SD	t-score
Personal Interest						
1	I desire to help others	4.32	±0.74	4.19	±0.71	4.117**
2	I can contribute to the society	4.24	±0.78	4.23	±0.67	0.48
3	I desire a fulfilling career	4.43	±0.71	4.15	±0.72	8.735**
4	I enjoy interacting with people	4.08	±0.84	4.17	±0.72	−2.591**
5	I want to make a difference in someone’s life	4.4	±0.70	4.27	±0.68	4.108**
Prior healthcare exposure						
6	In taking care of a sick family member	2.67	±1.22	3.59	±0.92	−18.248**
7	In being taken care of by a healthcare professional	2.7	±1.27	3.42	±0.95	−14.084**
8	In my school’s co-curricular activities	2.44	±1.20	3.44	±0.93	−19.876**
9	In observing healthcare professionals at work	3.35	±1.21	3.53	±0.89	−3.497*
10	In hearing about the profession from significant others	3.34	±1.11	3.66	±0.84	−6.829**
11	Doing voluntary work in healthcare settings	3.3	±1.18	3.84	±0.74	−11.272**
Self-efficacy						
12	Reflects well on academic ability	2.89	±1.11	3.17	±1.01	−5.454**
13	Choosing a course that is more deserving of grades	2.82	±1.17	3.04	±1.02	−4.424**
14	Can make autonomous decisions at work	3.3	±1.02	3.13	±1.01	3.730**
15	Wants to be able to make diagnoses	3.45	±1.04	2.95	±1.07	9.847**
Nature of the work						
16	It is a highly skilled occupation	3.7	±0.99	3.62	±0.86	1.577
17	I want a more hands-on job	3.96	±0.92	4.1	±0.74	−3.49*
18	It is a challenging job	3.73	±0.95	4.01	±0.81	−6.789**
19	It is a demanding job	3.31	±1.02	3.87	±0.90	−12.648**
20	I do not mind attending to others’ hygiene needs	3.34	±0.99	4.04	±0.80	−15.636**
Job prospects						
21	It ensures a stable job	3.73	±0.94	3.77	±0.78	−1.01
22	I will never be unemployed	3.27	±1.13	3.63	±0.93	−7.772**
23	It ensures high income	2.95	±1.08	2.89	±0.95	1.204
24	The career ensures me a good standard of living	3.32	±0.98	3.26	±0.89	1.323
25	It provides a chance to work overseas	2.87	±1.10	3.06	±1.00	−4.092**
26	It provides many opportunities for my career advancement	3.3	±1.01	3.4	±0.90	−2.086*
27	It provides a chance to achieve higher qualifications	3.39	±1.00	3.4	±0.86	−0.201
Social influences						
28	I will be well respected	3.22	±0.99	3.52	±0.91	−6.428**
29	It has a good public image	3.25	±0.99	3.64	±0.85	−8.555**
30	The social media has inspired me	2.62	±1.15	3.17	±1.02	−12.106**
31	There is no gender stigma in this career	2.91	±1.08	2.73	±1.18	3.554**
32	My parents are supportive	3.86	±0.95	3.54	±0.84	8.046**
33	I want my parents to be proud of me	3.71	±1.03	3.6	±0.80	2.494*
34	My peers encouraged me of my choice	3.31	±1.04	3.35	±0.84	−0.927
35	My peers will look up to me	2.77	±1.04	3.47	±0.83	−14.750**

#### Personal interest

As shown in Fig. 1, the overall mean scores for the subscale ‘personal interest’ was significantly lower (*p* < 0.001) in NCC than HCC, indicating that the participants are strongly influenced by the factor personal interest in making their career choice, but perceived this factor to have lesser influence on nurses’ choice of career. In Table 2, a further analysis among the different healthcare groups showed that this finding was only consistent among the medicine and dentistry students who rated NCC significantly lower (*p* < 0.001) than HCC. No significant differences were found between the HCC and NCC mean scores among pharmacy and social science students. Within this subscale, the item ‘desire for a fulfilling career’ showed the greatest disparity with significantly lower (*p* < 0.001) mean scores in NCC than in HCC.

#### Prior healthcare exposure

The overall mean scores for the subscale ‘prior healthcare exposure’ was significantly higher (p < 0.001) in NCC than HCC, suggesting that nursing as a career was perceived as more likely influenced by prior healthcare exposure. This finding was reported by students across all the different healthcare courses. Among the items in this subscale, the greatest disparity was found in the item ‘school co-curriculum activities’, where it was rated significantly higher in (*p* < 0.001) NCC than HCC.

#### Self-efficacy

There was no significant difference found between the overall self-efficacy mean scores for NCC and HCC and this finding was consistent across the different healthcare courses. However, there were significant differences in the mean scores for individual items. The items ‘reflects well on academic ability’ and ‘choosing a course that is more deserving of grades’ were rated significantly higher (*p* < 0.001) in NCC than in HCC, whereas the items ‘autonomous decisions at work’ or ‘can make diagnoses’ were rated significantly lower (p < 0.001) in NCC than in HCC. These findings suggested that the consideration of the ability to make autonomous decisions at work and the ability to make diagnoses have more influence on their healthcare career choice than nursing as a career choice.

#### Nature of the work

The overall mean scores for the subscale ‘nature of the work’ was significantly higher (*p* < 0.001) in NCC than HCC. This finding was only found to be consistent among pharmacy and social science students. Within this subscale, the item ‘do not mind attending to others’ hygiene needs’ was found to have the greatest disparity (p < 0.001), suggesting that this is an important part of nurses’ work but seems to be less desirable in healthcare students’ career choices.

#### Job prospects

The overall mean scores for the subscale ‘job prospects’ was significantly higher (*p* < 0.01) in NCC than HCC. This finding was only reported among students from the social science course. In contrast, the mean scores reported by the dentistry students were significantly lower (*p* < 0.05) in NCC than HCC. No significant difference was reported in the other healthcare courses. Within this subscale, the mean scores for the items ‘will never be unemployed’ and ‘a chance to work overseas’ were reported to be significantly higher (*p* < 0.001) in NCC than in HCC.

#### Social influences

The subscale ‘social influences’ was rated significantly (*p* < 0.001) higher in NCC than HCC. This suggests that students perceived nurses as more likely influenced by social factors to join nursing as compared to their own career choice. With the exception of dentistry, this finding was found to be consistent among all healthcare groups. Within this subscale, the items ‘well respected’, ‘good public image’, ‘inspired by social media’, and ‘peers will look up to me’ were rated significantly higher (p < 0.001) in NCC than in HCC. In contrast, the items ‘no gender stigma’, ‘parents are supportive’, and ‘want parents to be proud’ were rated significantly lower (*p* < 0.05) in NCC than in HCC.

## Discussion

This cohort of healthcare students was found to be strongly motivated by their personal interests in joining their healthcare careers. A similar finding has occurred in healthcare students across different countries such as Ireland [21] and the United States [22]. In our study, medical and dentistry students perceived their own personal interest as a stronger motivator to their career choice when compared to the personal interest of nurses. In Singapore as elsewhere, entry into medicine and dentistry requires a more rigorous and competitive recruitment selection criteria assessment also involving interviews to assess personal qualities [23]. With strong personal interests towards medicine or dentistry, these students were determined to pursue their desired career and were less likely to consider careers such as nursing. Given the importance of students’ personal interests in their chosen professions, nursing education institutions need to consider how to appeal to students through promoting the professional altruistic values and unique characteristics of nurses [24].

Students from all healthcare groups perceived that secondary students’ degree of prior healthcare exposure might have influenced their choice of nursing. A lack of nursing exposure could be a strong reason why these students did not pursue nursing as a career. Many studies have provided evidence that experiences such as taking care of others [25] or participating in hospital attachments or nursing camps [26] encouraged students to join the nursing profession. Our study found that schools provide sound platforms for developing a nursing interest among students. The implementation of caregiving programs within the school curriculum may help students to cultivate empathetic values, self-reliance, and respect towards the elders [27]. With the global aging population, caregiving programs could also motivate the younger generation to care for their older parents or grandparents at home. This may in turn help adolescents to develop an interest towards nursing [28]. Future studies could further explore the impact of prior exposures to career decisions through caregiving programs.

In this study, self-efficacy referred to a set of self-beliefs about one’s academic and intellectual abilities to a career choice. Surprisingly, non-nursing students recognized nurses’ academic abilities as a motivating factor to join the nursing career. This finding contradicts with several international studies from Greece, the United States, and the United Kingdom which found that students perceived nursing as less academic than other health professions [12, 13, 29]. One possible explanation could be the awareness of our participants on the availability of the nursing course offered in a renowned university in Asia. This may suggest that offering more nursing courses at prestigious universities may help to eliminate the misconception of nursing as a course for students with low academic performances especially within an Asian context.

Despite recognizing nurses’ academic abilities, the non-nursing healthcare students perceived nurses as less autonomous or have minimal diagnostic abilities compared to their own career choices. These findings are not new as several other studies also reported that students tend to perceive nurses as subservient to physicians and only follow doctors’ orders [10, 29]. As according to Dante et al. (2014), inaccurate understanding of the nursing career can increase the likelihood of students choosing courses other than nursing [30]. Hence it is important to remove such misconceptions through highlighting the clinical reasoning abilities of nurses. One way is through promoting the role of an advanced practice nurse who possesses a high degree of autonomy, and has the ability to make advanced health assessments with good decision making and diagnostic reasoning skills [31]. That way, students may be attracted to join nursing with the goal of becoming an advanced practice nurse.

Other deterring factors from a nursing career include the nature of nurses’ work being perceived as more hands on, challenging, and demanding in comparison to the students’ own career choices. Having to attend other’s hygiene needs’ was found to be the strongest deterring factor from the nursing career in this Singaporean based study. This was also a significant problem evident in the international literature [10–12]. The task of performing hygiene care was once considered ‘sacred’ in encouraging nurse-patient intimacy to allow a comprehensive patient assessment which in turn guides a patient-focused care plan [32]. The provision of care is an essence of nursing in providing comfort, wellbeing, and dignity to an individual [33]. Strategies need to be considered to help the younger generation overcome the fear of bodily fluids and see providing hygiene care as part of professional and valued societal work. Students in primary school can be exposed to simple hygiene care and gain understanding in why these activities were thought to be respected and important through the implementation of a caregiving program within the school curriculum.

Nursing was also seen as a career with considerable engendered stigma. This finding is unsurprising as male nurses remain a minority among the nursing population worldwide, and in some countries are perceived to be negatively associated with a particular sexual orientation. [34]. Within the Asia context, nursing continues to be seen as a woman’s occupation and, hence, posing a barrier to male recruitment and retention in the profession. Moreover, healthcare students in our Singaporean based study perceived nursing as a careers choice to not meet parental approval or consent. Studies have shown that parents play a significant role in their child’s career decision [35, 36]. This is especially true within the Chinese culture, in which filial piety and achieving good academic results are attributes of an ideal child [37]. In comparison, parents seem to have less influence over Western students who are more likely to make independent decisions on career choices [38]. A previous qualitative study done within Singapore found that students who were interested in nursing faced parental objection when making their choices [8]. The cultural importance of fulfilling filial duties in Asia means that, parents’ decisions tend to outweigh their children’s choices [39]. Therefore, government agencies promoting nursing as a career choice should not only target the younger generations, but also ensure that parent receive accurate information on nursing career progression. Future studies could look into exploring parents’ perceptions of their children joining the nursing profession and determine how students who are interested in nursing could be better supported.

### Limitations

The study findings may be limited by its generalizability as convenience sampling method was used. Students completed the questionnaire on voluntary basis, and those who participated could have had a more positively orientated perception of nursing. Nevertheless, this limitation has been reduced by recruiting the total population of potential participants. This study may also be limited by population selection bias as the participants have already started their study in a healthcare course and are likely more positive on their own career. If possible, recruiting these students prior to course selection would reduce such biasness. The findings may also be limited in its transferability as it involved a single site study conducted in Singapore. However, as nursing workforce shortages pose a global problem, we would content that these findings have international significance.

## Conclusion

This study suggests that university students may be less likely to choose nursing largely due to a lack of healthcare exposure, their dislike in dealing with hygiene needs, a lack of autonomous decision making, gender stigma, and a lack of parental support. Healthcare education institutions and policy makers could use this information to consider strategies in making the nursing profession more attractive to school leavers:

Firstly, opportunities should be created for exposure to the work of health professionals, in particular nurses to students during early school years. These could be in the form of hospital visits, community elderly befriending, caregiving courses, or nursing camps.

Secondly, there is a need to help younger people overcome the fear of handling bodily fluid and personal hygiene care. Caregiving courses could be incorporated into school curriculum to increase exposure on hygiene care, to help students cultivate self-care independence and to improve their ability to care for their elders at home.

Thirdly, there is a need to promote autonomous decision making among nurses by recognizing their critical thinking skills and leadership abilities. More attention should be delegated to the general public through media campaigns, identifying registered nurses as professionals to better inform the perception of nursing as a respected and progressive career.

Fourthly, there is a need to promote male student entry into nursing. This could be done through publicizing the success stories of male nurses holding key leadership positions as well as making the nursing curriculum more attractive to male students.

Lastly, it is important that parents see the nursing career as a profession that requires a high level of skills and knowledge to provide quality care comparable with other professions. Parents need to be strongly encouraged at the school level to attend nursing career talks or hospital attachments with their sons or daughters to better understand nursing as a career. With sound knowledge of the career progression, it is less likely that Asian parents would restrict their children’s choices in nursing.
